# Hinge like domain motion facilitates human RBMS1 protein binding to proto-oncogene *c-myc* promoter

**DOI:** 10.1093/nar/gkab363

**Published:** 2021-05-17

**Authors:** Priyanka Aggarwal, Neel Sarovar Bhavesh

**Affiliations:** Transcription Regulation group, International Centre for Genetic Engineering and Biotechnology (ICGEB), Aruna Asaf Ali Marg, New Delhi 110067, India; Transcription Regulation group, International Centre for Genetic Engineering and Biotechnology (ICGEB), Aruna Asaf Ali Marg, New Delhi 110067, India

## Abstract

DNA binding proteins recognize DNA specifically or non-specifically using direct and indirect readout mechanisms like sliding, hopping, and diffusion. However, a common difficulty in explicitly elucidating any particular mechanism of site-specific DNA-protein recognition is the lack of knowledge regarding target sequences and inadequate account of non-specific interactions, in general. Here, we decipher the structural basis of target search performed by the key regulator of expression of *c-myc* proto-oncogene, the human RBMS1 protein. In this study, we have shown the structural reorganization of this multi-domain protein required for recognizing the specific *c-myc* promoter sequence. The results suggest that a synergy between structural re-organization and thermodynamics is necessary for the recognition of target sequences. The study presents another perspective of looking at the DNA-protein interactions.

## INTRODUCTION

Protein-DNA interactions are the choreographers of cellular processes ranging from as basic as the chromosomal organization to as complicated as translation (1). They are responsible for maintaining the integrity of the genome as well as controlling all major and minor cellular mechanisms (2,3). It is imperative that the DNA binding proteins locate their specific DNA targets in the highly dense nucleus of the cell (4). The functioning of DNA binding proteins relies on locating their precise DNA targets through stochastic search processes. For achieving this, they bind a range of non-specific sequences, scan and recognize them by combining 1D sliding, hopping, and 3D diffusion and ultimately reach their specific targets to perform their designated functions (5). There is a symphony of specific/non-specific DNA–protein interactions at the heart of the cell, which master-regulates all the cellular processes. The network of protein–nucleic acid interactions inside a eukaryotic cell is very complicated, far away from the oversimplification of specific and non-specific interactions. The atomic resolution structural and mechanistic studies of protein-nucleic acid complexes are difficult to conduct without the prior knowledge of consensus sequences.

Human RNA Binding Motif Single Stranded Interacting Protein 1 (RBMS1) is one such protein that was first isolated in 1994 as one of the family of *myc* gene single stranded binding proteins and has been shown to control the expression of proto-oncogene *c-myc* inside the human cell. c-myc protein is a transcription factor that binds both specifically and non-specifically to activate the transcription of several downstream gene targets and therefore, even minor fluctuations in c-myc levels have profound effects on cellular growth and transformations (6). RBMS1 contains two most abundantly present nucleic acid binding domains in the eukaryotes, the RNA recognition motifs (RRMs also known as ribonucleoprotein (RNP) domain), with two highly conserved submotifs—octameric RNP-1 ((R/K)-G-(**F**/**Y**)-(G/A)-(**F/Y**)-V-X-(F/Y)) and hexameric RNP-2 ((L/I)-(**F**/**Y**)-(V/I)-X-(N/G)-L) within each of the domains (7,8). RBMS1 stimulates DNA replication, transcriptional regulation, and cell transformation by specifically binding to the 7 bp consensus sequence A/TCTA/TA/TT within the 21 bp promoter sequence/autonomous origin of replication 2 kb upstream of *c-myc* gene (9–11).

The mechanism of *c-myc* promoter recognition by RBMS1 is not known at the molecular level. In this study, we report the structural and thermodynamics basis of the DNA binding mechanism of RBMS1. We have delineated the structural basis of specific recognition of *c-myc* promoter by RBMS1 protein with the help of the three-dimensional structures of RBMS1 in free and *c-myc* promoter DNA bound states determined in this study. This study provides a deeper understanding of the mechanism that is followed by exclusive and stringent DNA promoter binding proteins during the stochastic DNA search process.

## MATERIALS AND METHODS

### Cloning, expression and purification of RBMS1 protein

The coding sequence of RBMS1 (Uniprot Id P29558) was optimized for expression in *Escherichia coli*. The clone was synthesized from GeneArt (Life technologies). The bioinformatics software predicted the boundary of the second RRM domain till 219 amino acid residues only and hence, the construct initially was cloned from 58–219 amino acid residues in the pETM11 vector for protein expression and the recombinant protein was purified. The 2D [^15^N, ^1^H] HSQC spectrum, however, showed severe line broadening and overlap of resonance peaks. When the construct boundary was increased by the addition of five amino acid residues at the C-terminal end, the 2D [^15^N,^1^H] HSQC spectrum showed a very well folded and stable protein with much less line broadening and resonance overlap (Supplementary Figure S1). The dramatic changes seen in the NMR spectra helped us in correctly determining the domain boundaries. The subclone was then prepared corresponding to the amino acid residues from 58–224. Primers used for amplification were 5′ GCGCCATGGGAACCAATCTGTATATTCGTGGTCTGCCT 3′ forward primer and 5′ GCGCTCGAGCTAATCCTGTTCTTGCTGTTTTGCCAT 3′ reverse primer. The cloning of the construct was done in the expression vector pETM11, downstream of 6X Histidine-Tag cleavable by tobacco etch virus (TEV) protease. The vector plasmid was transformed for protein expression into *Escherichia coli* BL21(DE3) *CodonPlus* cells. The cloned genes were verified by sequencing (Macrogen, Inc.).

For expression of recombinant protein, *E. coli* bacterial cells were grown up to an OD_600_ of ∼0.8–1 in Luria–Bertani broth. The culture was induced using 0.5 mM IPTG at 25°C for 16–20 h. Cells were collected by centrifugation at 3584xg rcf for 20 min, lysed by sonication by resuspension in a binding buffer consisting of 20 mM sodium phosphate (pH 7.1), 300 mM NaCl, 5% (*v/v*) glycerol and 10 mM imidazole. The sample was added to Ni-NTA affinity chromatography resin (Qiagen) and was washed with 20 column volumes of the same binding buffer but with 20 mM imidazole. Elution was done in buffer with 20 mM sodium phosphate (pH 7.1), 300 mM NaCl, 5% (*v/v*) glycerol and 300 mM imidazole followed by cleavage with TEV protease for 16 h at 20 °C. The TEV protease, 6X-His tag, and the uncleaved protein were removed by again performing Ni-NTA affinity chromatography, followed by size exclusion chromatography using S75 16/60 GE column, in the buffer containing 20 mM sodium phosphate (pH 7.1) and 100 mM NaCl. The centrifugal filters (Merck, Millipore) with 3000 Daltons molecular weight cutoff was used for the concentration of fractions up to ∼1 mM. Protease inhibitor cocktail (Roche) was added to the final protein preparation and storage of the protein aliquots was done at −80 °C.

The preparation of isotopic labeled *U*-^15^N or *U*-^13^C,^15^N-labeled recombinant proteins was done using 2.5 g/L ^13^C_6_–d-glucose and 1.0 g/l ^15^NH_4_Cl (Cambridge Isotope Laboratories), as sole carbon and nitrogen sources, respectively in M9 minimal media, yielding the uniformly ^13^C,^15^N-labeled protein. Growth in M9 minimal medium yielded about 100 mg of pure RBMS1 (58–224) from 1 liter of culture. The cleavage of 6x-His tag was done using TEV protease before performing size exclusion chromatography that left the tag related tetrapeptide GAMG at the N-terminal of the protein. The NMR buffer used for final protein preparations consisted of 20 mM sodium phosphate pH 7.1, 100 mM NaCl and 5% D_2_O (*v/v*).

### Cloning, expression and purification of isolated RRM1 and RRM2 domains of RBMS1

The two sub clones corresponding to RRM1 (amino acid residues 58–137) and RRM2 (amino acid residues 138–224) domains were also made. Primers used for amplification were 5′ GCGCCATGGGAACCAATCTGTATATTCGTGGTCTGCCT 3′ forward primer and 5′ GCG CTCGAG CTA ATC CTG TTC TTG CTG TTT TGC CAT 3′ reverse primer for RRM1, and 5′ ‘GCG CCATGG GA ACAAACCTGTATATTAGCAATCTGCCG’ 3′ forward primer and 5′ GCGCTCGAGCTAATCCTGTTCTTGCTGTTTTGCCAT 3′ reverse primer for RRM2. The cloning of both the constructs was done into the expression vector pETM11, downstream of 6X His tag, cleavable by tobacco etch virus (TEV) protease. The vector plasmid was transformed for protein expression into *E. coli* BL21(DE3) *CodonPlus* cells. The cloned genes were verified by sequencing (Macrogen, Inc.). The purification protocol was the same as the one that was followed for the RBMS1 (58–224) construct.

### Oligonucleotides

DNA oligonucleotides used as ligands were purchased from Sigma-Aldrich in the desalted form. In order to understand the specificity of DNA, we designed 29 different DNA sequences, in which either one of the bases of the seven nucleotide consensus binding DNA sequence from *c-myc* gene promoter, i.e. TCTTATT was randomly changed to any of the other three bases. Some sequences were designed in a way to keep the TAT core sequence similar and change the other one or more bases to see the effect of nucleotide substitution on protein binding. Other sequences were designed in a way that the core sequence was not retained and other combinations from the 5'-3' promoter or its complementary sequence were tested for their binding to the protein. Rest sequences were designed to check the specificity of binding for the length of the sequence, we took just the core sequence TAT and any random base at the start to see if the core was still recognized by the protein.

### Site directed mutagenesis

Protein mutants (Y105S, F107L, Q135E and F185V) were generated by site directed mutagenesis using a set of internal PCR primers that contained the mutated sequence. The mutant plasmids were verified by DNA sequencing. Mutant proteins were expressed and purified by using methods similar to those used for the wild type protein. The folding of protein mutants was confirmed using NMR spectroscopy. The primers used for each mutagenesis are shown in Supplementary Table S4.

### Solution-state NMR spectroscopy

Samples contained 1 mM RBMS1 protein in 20 mM sodium phosphate pH 7.1, 100 mM NaCl and 5% D_2_O (*v/v*). NMR experiments were conducted on a Bruker Avance III equipped with a 5 mm cryogenic triple resonance TCI probe-head, operating at the field strength of 500.15 MHz at 303 K. Spectra were processed using Topspin 3.1 (Bruker AG) and analyzed using Computer Aided Resonance Assignment (CARA) software (12). The experiments (13) used for protein resonance assignment were standard double and triple resonance spectra, namely, 2D [^15^N,^1^H]-HSQC, 2D [^13^C,^1^H]-HSQC [aliphatic (0 to 5 ppm ^1^H_ali_) and aromatic (4.7 to 10 ppm ^1^H_aro_)], 3D CBCAcoNH, 3D HNCA, 3D HNCO, 3D HNCACB, 3D HcccoNH, 3D hCccoNH,; all of them were acquired at 303 K. For calculation of distance restraints, a set of NOESY spectra (NOESY mixing time of 100 ms), namely, 3D ^15^N-edited [^1^H,^1^H]-NOESY at 500.15 MHz spectrometer, 3D ^13^C_ali_-edited [^1^H,^1^H]-NOESY in H_2_O at 500.15 MHz spectrometer and in D_2_O at 800.18 MHz spectrometer, and 3D ^13^C_aro_-edited [^1^H,^1^H]-NOESY at 500.15 MHz spectrometer were measured at 303 K.

Manual assignment of backbone and side-chain resonances was done using Computer Aided Resonance Assignment (CARA) software with ^1^H shifts calibrated with respect to 2,2-dimethyl-2-silapentane-5-sulfonate (DSS) at 303 K (0.0 ppm). ^13^C and ^15^N chemical shifts were referenced indirectly to the DSS methyl proton resonance at 0 ppm in all spectra. TALOS-N was used for deriving backbone (φ, ψ) and side-chain (χ1) dihedral angle from the observed chemical shifts (14).

### Solution structure calculation using NMR spectroscopy

For calculation of the solution structure, the cross-peaks in the NOESY spectra were used to derive the inter-proton restraints up to a limit of 5 Å. NOE intensities were used for classification of distances as 1.8- 2.4 Å (strong), 1.8–3.5 Å (medium), 1.8–5.0 Å (weak). A total of 2185 distance constraints (around 17 per residue) were used for structure calculation using the program CYANA 3.98.13 (15), using distance geometry and simulated annealing protocol of 20 000 steps. Further refinement of the top 20 cyana structures with the least residual target function and violations was done by simulated annealing and energy minimization in explicit solvent using the SANDER module of the AMBER18. The amber ff14SB force field (16) was used for the minimization. The final ensemble comprised of 20 structures with the lowest energy.

### Backbone ^15^N relaxation experiments

Backbone nuclear spin relaxation (μs-ps dynamics) of RBMS1 in free and DNA bound form were measured using ^15^N–{^1^H}-heteronuclear nOe and ^15^N T1, T2 relaxation experiments using Echo/Anti-echo-TPPI gradient selection as pseudo 3D. Sixteen delays ranging from 20 to 1000 ms were used for T1, while 14 loop counters for the CPMG pulse train were set to get T2 delays from 10 to 210 ms. The ^15^N–{^1^H} heteronuclear nOe were measured using the pulse sequence hsqcnoef3gpsi3d. The ratio of peak intensities with and without a 4 s proton saturation was used to obtain the steady-state ^15^N–{^1^H} nOe values. A recycle delay set to 2.5 s allowed the ^15^N and ^1^H spins to return to equilibrium. The spectra were processed using Topspin 3.1 (Bruker AG) and all the calculations were done using the Dynamics Center 2.5.4 (Bruker AG). The principal components of the anisotropic diffusion tensors were calculated using the ROTDIF 1.1 software (17). The residues whose ^15^N–{^1^H} Het-nOe values were less than 0.65 were excluded from the diffusion tensor calculations.

### NMR spectroscopy of protein–DNA complexes

To map the interface of protein–DNA complexes, titration of 0.5 mM *U*-^15^N protein was done against the molar ratios of DNA increasing in steps of 0.2 from 1:0 to 1:1.2. 2D [^15^N,^1^H] HSQC spectrum was recorded at each step and was used for tracking changes in chemical shifts (chemical shift perturbations, Δδ) of the backbone amide protons at each molar ratio. Calculation of CSPs was done using the equation –

$$\begin{equation*}\Delta {\rm{\delta }}{{\rm{ }}_{15}}_{{{\rm{N}}^{\rm{H}}},{{\rm{H}}^{\rm{N}}}}{\rm{ }} = {\rm{ }}\sqrt {{{\left( {\frac{{\Delta {\rm{\delta }}{{\rm{ }}_{15}}_{{{\rm{N}}^{\rm{H}}}}}}{5}} \right)}^2}{\rm{ }} + {\rm{ }}{{\left( {\Delta {{\rm{\delta }}_{{{\rm{H}}^{\rm{N}}}}}} \right)}^2}} \end{equation*}$$

where ΔδH^N^ and Δδ^15^N^H^ are the changes in backbone amide chemical shifts for ^1^H^N^ and ^15^N resonances, respectively.

### X-ray crystallography of protein–DNA complex

For co-crystallization, protein RBMS1 was mixed with DNA sequence TCTTATT in an equimolar ratio of 1:1 and was incubated at room temperature for 2 hours prior to setting up the crystal trays. The final protein concentration was 30 mg/ml in a buffer containing 20 mM HEPES pH 7.5, 50 mM NaCl, 10 mM MgCl_2_ and 10 mM β-Mercaptoethanol. The crystals were grown at 273 K by the hanging drop vapor diffusion method and the reservoir contained 0.05 M Magnesium Sulfate Hydrate, 0.05 M HEPES sodium pH 7.0, 1.6 M lithium sulfate and 30% methanol. The thin plate-shaped crystals were soaked in cryoprotectant paratone oil and were directly mounted in a stream of cooled nitrogen gas at 100 K. Cu Kα radiation (λ = 1.54 Å) at 100 K was used for the collection of X-ray diffraction data using a Rigaku FR-E+ SuperBright microfocus rotating-anode (dual-wavelength; Cu and Cr) X-ray generator that was equipped with an R-AXIS IV^++^ detector, operating at 45 kV and 55 mA. Oscillation steps of 0.5° were used to collect a total of 509 frames. The exposure time of each frame was kept 240 s. The diffraction images set was processed and scaled using the autoPROC package (18). The structure was solved using phaser-MR with HuD in complex with C-FOS RNA as a template (38% sequence identity, PDB: 1FXL). The initial model was built using AutoBuild in PHENIX (19) and was followed by multiple rounds of the manual model building using Coot (20) in combination with running refinement cycles in PHENIX. UCSF Chimera (21) and PyMol (http://www.pymol.org) softwares were used for all the structure visualizations and preparing images.

### Isothermal titration calorimetry

ITC experiments were conducted at 303 K in the GE MicroCal iTC200 calorimeter. ITC cell was filled with 0.1 mM protein and 1 to 1.5 mM DNA was filled in the syringe. Both protein and DNA were prepared in the buffer containing 20 mM sodium phosphate (pH 7.1) and 50 mM NaCl in filtered water. Protein and DNA concentrations were measured at 280 and 260 nm, respectively. Titrations consisted of sequential injections of DNA with the first injection of 0.4 μl followed by 39 injections of 1 μl volume. A 120 s interval was kept between the injections. The reaction mixture in the sample cell was constantly stirred at 750 rpm. To determine the change in enthalpy due to ligand dilution, titration of RBMS1 was performed with buffer alone. This was then subtracted as background from the actual DNA binding experiments. The results gave heats that were fitted to a one-site model using Origin 7 software.

### MD simulations

PDB files of RBMS1 and RBMS1-TCTTATT complex structures were prepared for Molecular Dynamics using the Desmond 3.1 MD package (Schrödinger Inc.). The molecule was placed inside an orthorhombic box to impose periodic boundary conditions, ensuring a solvent shell of at least 10 Å around the molecule, which was subsequently filled with water molecules using the TIP3P solvent model and was neutralized by the addition of Na^+^/Cl^–^ ion pairs to reach a concentration of 150 mM. Prior to simulation, the system was minimized for 100 ps. The simulation time was set to 1000 ns and the standard NPT ensemble system (isobaric-isothermal condition) was used for simulation wherein the constant temperature used was 300 K, and constant pressure 1.01325 bars under the force field OPLS3e (22). The co-ordinate frames were saved at intervals of 4.8 ps for analysis. The time step used was 2 fs. Input and output files were prepared, analyzed, and visualized using Maestro graphical user interface (GUI).

## RESULTS

### RRM domains of RBMS1 form globular structure and do not interact with each other

**Figure 1.**
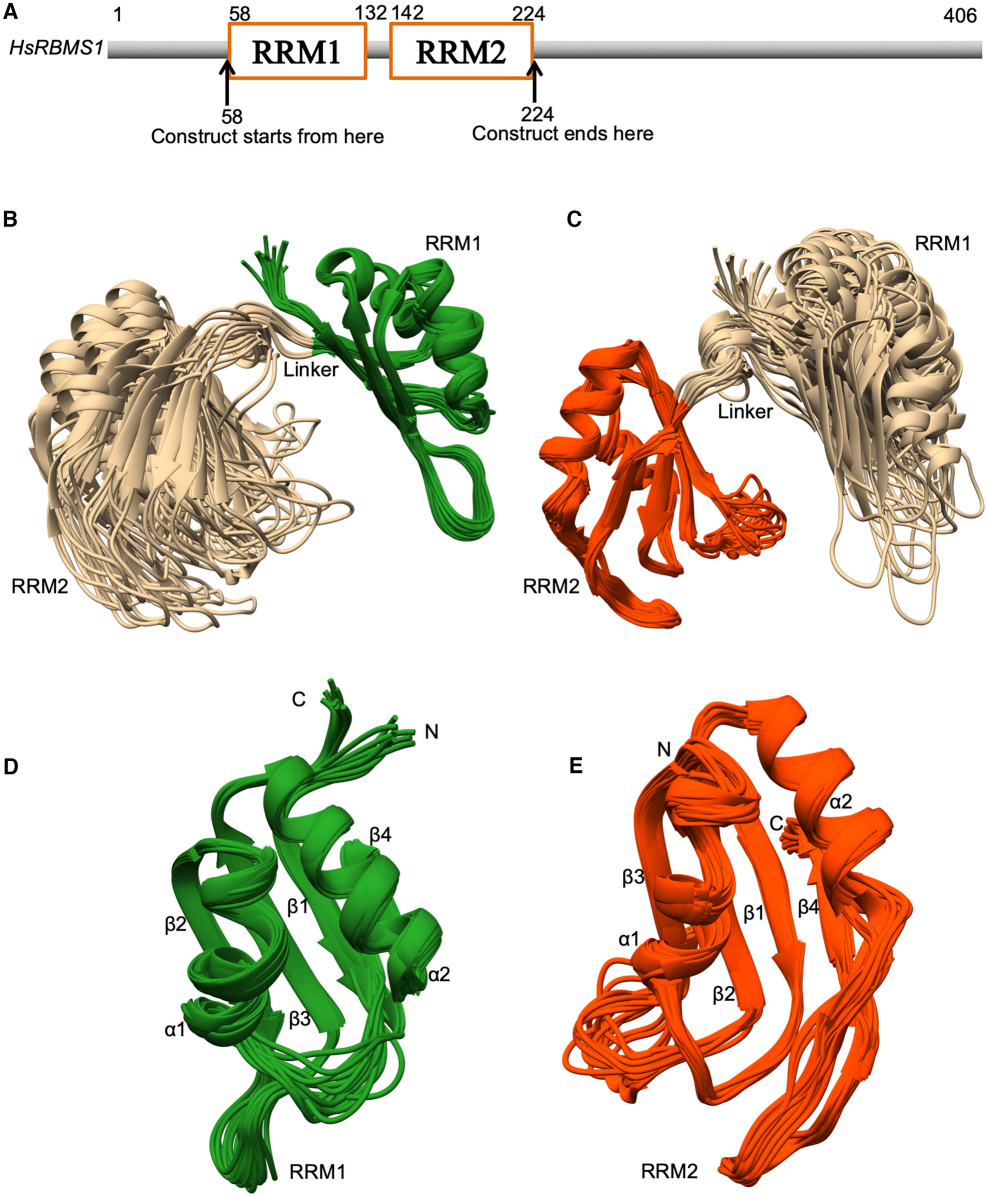
(**A**) Schematic of the domain architecture of RBMS1 protein showing the construct boundaries. (**B**) Superposition of the backbone atoms of the 20 energy-minimized conformers of RBMS1 as aligned with respect to the RRM1 domain (residues 58–132). (**C**) Superposition of the backbone atoms of the energy-minimized conformers of RBMS1 aligned with respect to the RRM2 domain (residues 142–224). (**D**) Superposition of the RRM1 domain of RBMS1. (**E**) Superposition of the RRM2 domain of RBMS1. Secondary structural elements, N- and C-terminals are marked.

The domain architecture of human RBMS1 protein comprised of two RRM domains separated by a stretch of only nine amino acid residues between them (Figure 1a). After the optimization of protein construct boundaries (see Materials and Methods), protein (58–224) was found to exist as a monomer by size exclusion chromatography (Supplementary Figure S1). The purified homogeneous RBMS1 protein was used to obtain complete sequence-specific NMR assignments and calculate solution structure. A superimposition of 20 lowest energy structures of RBMS1 (PDB id 7C36) is shown in Figure 1. The NMR structural parameter statistics for the energy-minimized 20 conformers of RBMS1 was calculated using cyana 3.98.13 and are given in Supplementary Table S1. The solution NMR structure within the two domains was very well defined separately and good convergence was seen within each of the domains. The superimposition of the conformers when aligned with respect to the RRM1 domain (amino acid residues 58–132) and when aligned with respect to the RRM2 domain (amino acid residues 142–224) are shown in (Figure 1B and C). Both the domains had the canonical RRM fold of β1–α1–β2–β3–α2–β4; with the two α-helices packed against an antiparallel four stranded β-sheet. The superimposition of the RRM1 and RRM2 domain has been shown separately in Figure 1d and e, respectively. The canonical RNA-binding ribonucleoprotein (RNP) sites, RNP1 and RNP2 were conserved (Supplementary Figure S2) and present on the β3 and β1 strands of both the domains, respectively. A highly flexible linker of 9 amino acid residues (amino acid residues 133–141) connected the two domains and resulted in spatial heterogeneity of the domains about this region. Poor convergence of structures in this region was attributed to a small number of nOes observed for the residues in the linker region due to conformational averaging. The flexibility in the linker region was supported by the secondary chemical shifts and NMR relaxation parameters for the residues in the region (Supplementary Figures S2b and S3). RRM1 and RRM2 domains did not interact with each other in the free form of the protein and this was also evident from the overlay of 2D [^15^N,^1^H] HSQC spectra of RRM1 domain (58–137) and the RBMS1 protein (58–224) as well as from the overlay of 2D [^15^N,^1^H] HSQC spectra of RRM2 domain (138–224) and the RBMS1 protein (58–224) in which no CSP were observed in the resonances of the amide protons of the RRM1 domain and RRM2 domain, respectively and all of them overlapped completely with those in the 2D [^15^N,^1^H] HSQC spectrum of the protein (58–224) (Supplementary Figure S4). The flexible linker resulting in the independent domain motion could be one of the possible reasons that our attempts to obtain crystals of RBMS1 did not succeed.

### Both RRM domains of RBMS1 are required for DNA binding

**Figure 2.**
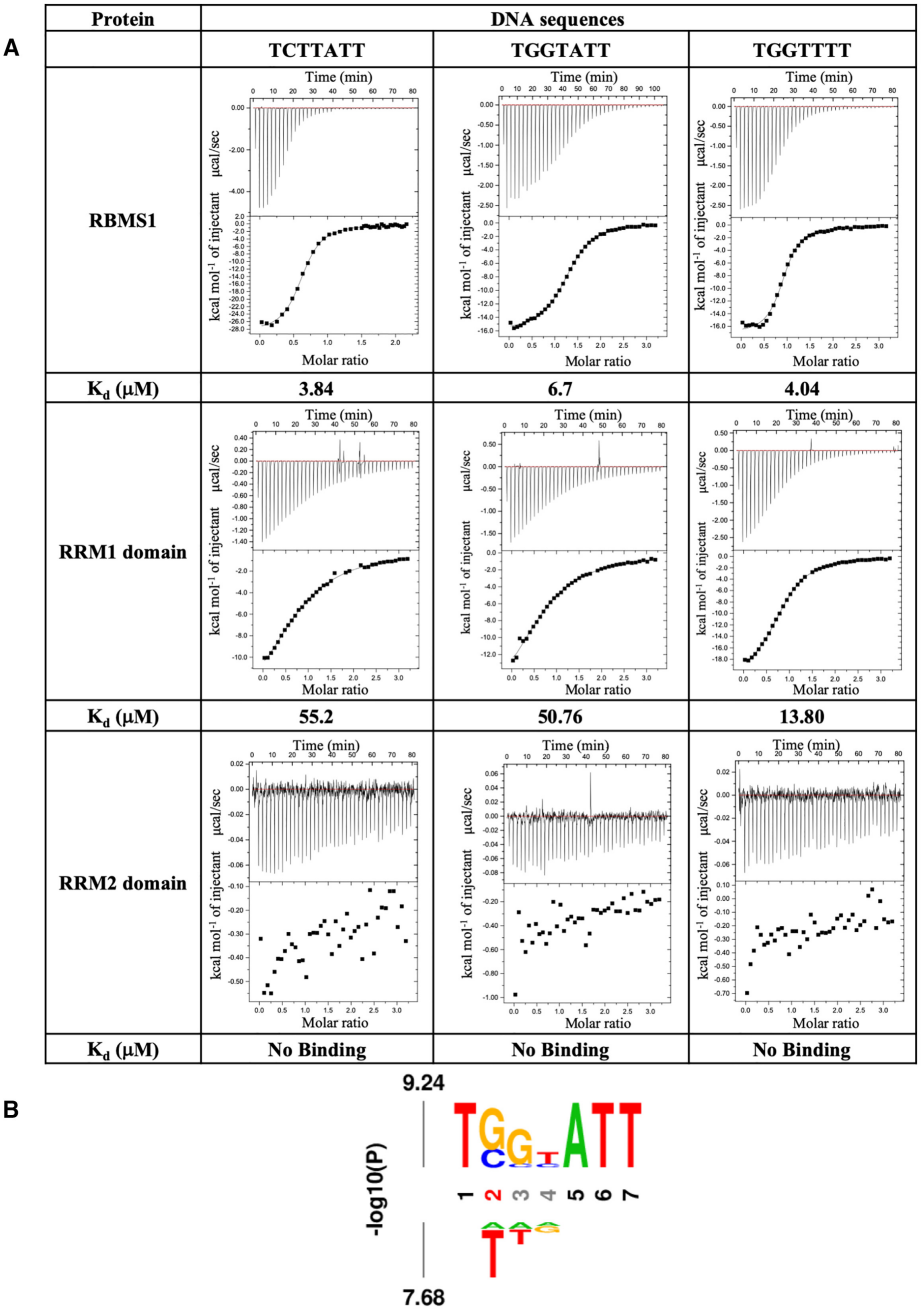
(**A**) ITC thermograms and affinity (K_d_) values of the selected DNA sequences with the RBMS1 protein (58–224) and the two domains individually. The RRM2 domain alone does not show binding with any of the DNA sequences. (**B**) Relative affinity of nucleotide substitutions within the cognate *c-myc* promoter DNA sequence TCTTATT ligand calculated using ITC. The height of the A (adenine), C (cytosine), G (guanine) and T (thymine) is relative to the determined values of the dissociation constant, *K*_d_ (Supplementary Table S2).

The truncated RBMS1 protein containing both RRM domains (58–224) was used for DNA binding studies. The protein interacted with the full-length 21 bp promoter sequence of *c-myc* with the affinity of 2.6 μM and to the seven nucleotides consensus sequence TCTTATT within the full-length promoter sequence with an almost similar affinity of 3.84 μM in the ITC experiment (Supplementary Table S2). Hence, in order to further understand the role of individual RRMs in the promoter DNA binding, we made two more constructs of the individual domains RRM1 (amino acid residues 58–137) and RRM2 (amino acid residues 138–224), which were again purified to homogeneity (Supplementary Figure S1). The selected consensus sequence from the upstream *c-myc* gene promoter sequence TCTTATT was titrated against both the shorter domain constructs of RBMS1 protein, i.e. RRM1 domain, and RRM2 domain. While the RRM2 domain showed no binding with the promoter sequence, the binding affinity of the RRM1 domain decreased 18-fold from 3.84 μM with RBMS1 (58–224) to 55.2 μM (Figure 2A). The presence of both the domains was deemed necessary for interaction with the promoter DNA sequence.

### Minimum length of six nucleotides of *c-myc* promoter sequence having trinucleotide ATT at 3′ end is required for binding with RBMS1

In order to understand the specificity of DNA sequence interactions, we performed a series of ITC experiments with 29 different DNA sequences, in which the bases of the 7 nucleotide consensus binding DNA sequence from *c-myc* gene promoter i.e. TCTTATT were randomly changed to any of the other three bases. Also, in a few cases, more than one nucleotide base in the sequence was changed or where the length of the sequence was altered to have four or five nucleotides only. The entire list of DNA sequences, which were titrated for the binding studies and the thermodynamic parameters obtained with them are given in Supplementary Table S2. The sequence logo that was derived from the sequences and the dissociation constant values obtained with them is shown in Figure 2B. The affinity measurements using ITC showed that there is a sequence preference for molecular recognition by the RBMS1 protein but confounded the idea that there was any simple code of recognition. When the length of the sequence was reduced to four or five nucleotides only, the binding was abolished, showing that the protein preferred a minimum DNA length of six nucleotides for binding. Results also showed that the binding preference of the protein for some DNA bases at the given positions, for example, the substitution of C with G at the second position of 7 nucleotide DNA sequence, reduced the affinity of protein towards the resulting DNA sequence TGTTATT from 3.84 to 15.2 μM (Supplementary Table S2).

Although we analyzed a lot of different DNA sequences thermodynamically using ITC, a simple pattern of recognition was not seen for this protein. We, therefore, selected 3 out of all of these DNA sequences for further analysis; this included one specific consensus promoter sequence of *c-myc* gene, TCTTATT, and two other sequences, TGGTATT and TGGTTTT that showed good affinity. The rationale behind selecting these two sequences amongst others was that these DNA sequences showed higher affinity than the other sequences for the RBMS1 protein (58–224) in the ITC experiments. The three selected DNA sequences were then titrated against three constructs of RBMS1 protein, i.e. the RBMS1 (58–224), the RRM1 domain (58–137), and the RRM2 domain (138–224) (Figure 2a). There was no binding of any of the three DNA sequences with the RRM2 domain. The binding affinity of the TGGTATT and TCTTATT sequence decreased 10- and 18-fold, respectively with the RRM1 domain as compared to the RBMS1 protein (58–224).

In order to map specific residues on the protein surface, which were involved in binding to the DNA sequence, we performed NMR titration of the DNA sequences with RBMS1 (58–224) and calculated chemical shift perturbations (CSP). The common patches of amino acid residues involved in binding to the nucleic acid sequences fell mainly on the β strands of both the domains. In the RRM1 domain, amino acid residues on the β3 strand, such as T91, G104, Y105, while residues on β1 and β3 strands of the RRM2 domain such as T141, L161, F185 showed significant perturbation, indicating that these are the main residues involved in nucleic acid binding (Supplementary Figure S5). Interestingly, the amino acid residues such as K134 and Q135 in the linker region (amino acid residues 133–141) also showed significant perturbations indicating their possible role in binding to the DNA sequence. Another interesting observation was that the binding of non-specific sequence TGGTTTT caused perturbations in the residues on RRM1 domain majorly, while the TGGTATT and TCTTATT sequences caused perturbations in residues on both the RRM1 and RRM2 domains.

### RBMS1 protein binds with *c-myc* promoter DNA in a non-canonical manner

**Figure 3.**
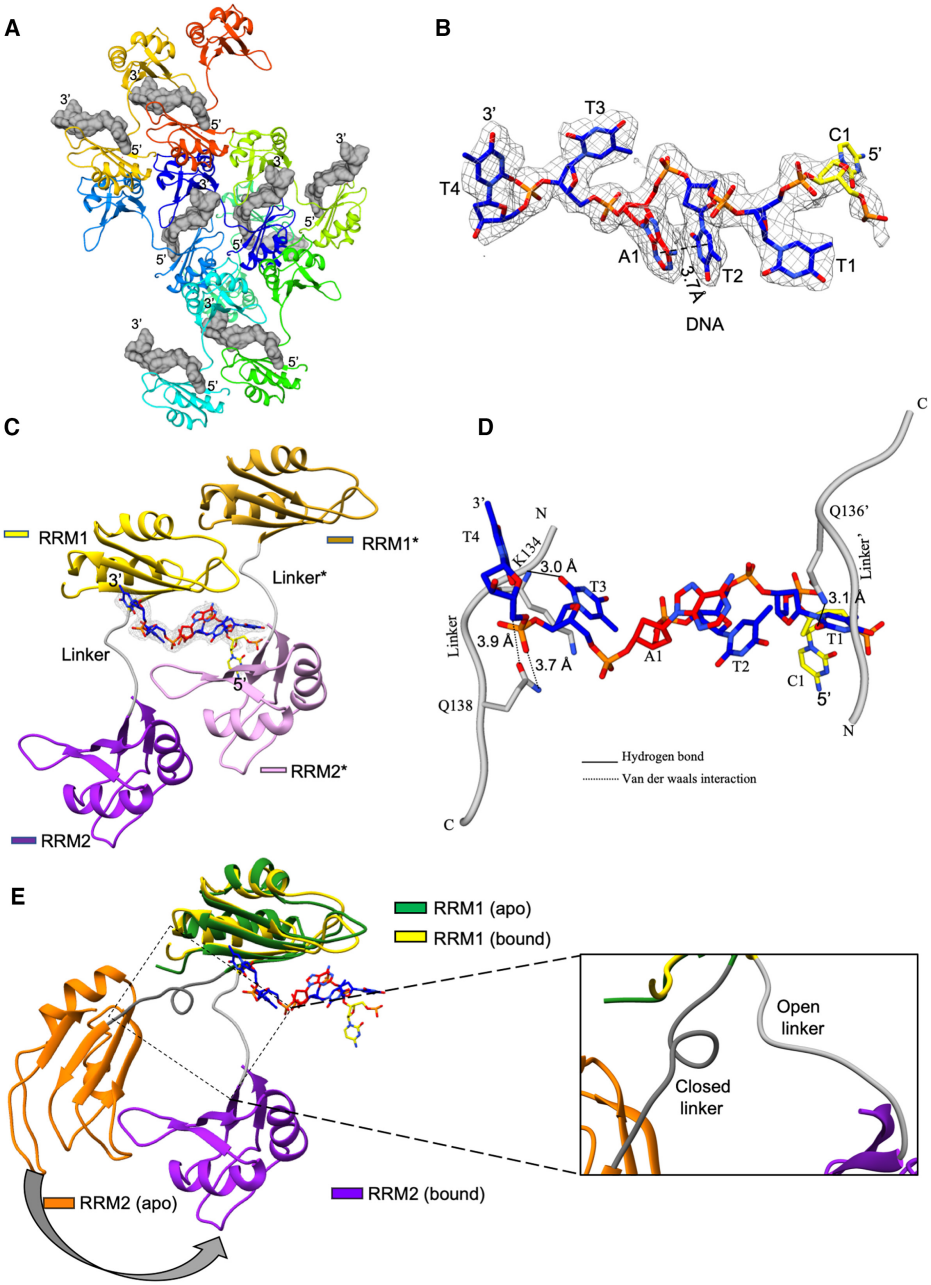
(**A**) X-ray structure of the *c-myc* promoter bound to RBMS1 protein (58–224) and its symmetry related molecules. (**B**) The electron density omit map of DNA at 1σ in the bound form with the stacking distance of 3.7 Å between A1 and T2 nucleotides is shown. (**C**) Zoomed in view of DNA binding between protein and one symmetry related molecule. (**D**) The interactions between the amino acid residues of the linker and the DNA nucleotides of promoter DNA sequence are shown. (**E**) Superimposition of the solution-state NMR structure of apo RBMS1 with the crystal structure of RBMS1 bound with DNA showing the opening of the 3_10_ helix in the linker region and movement of the RRM2 domain to bind DNA.

**Figure 4.**
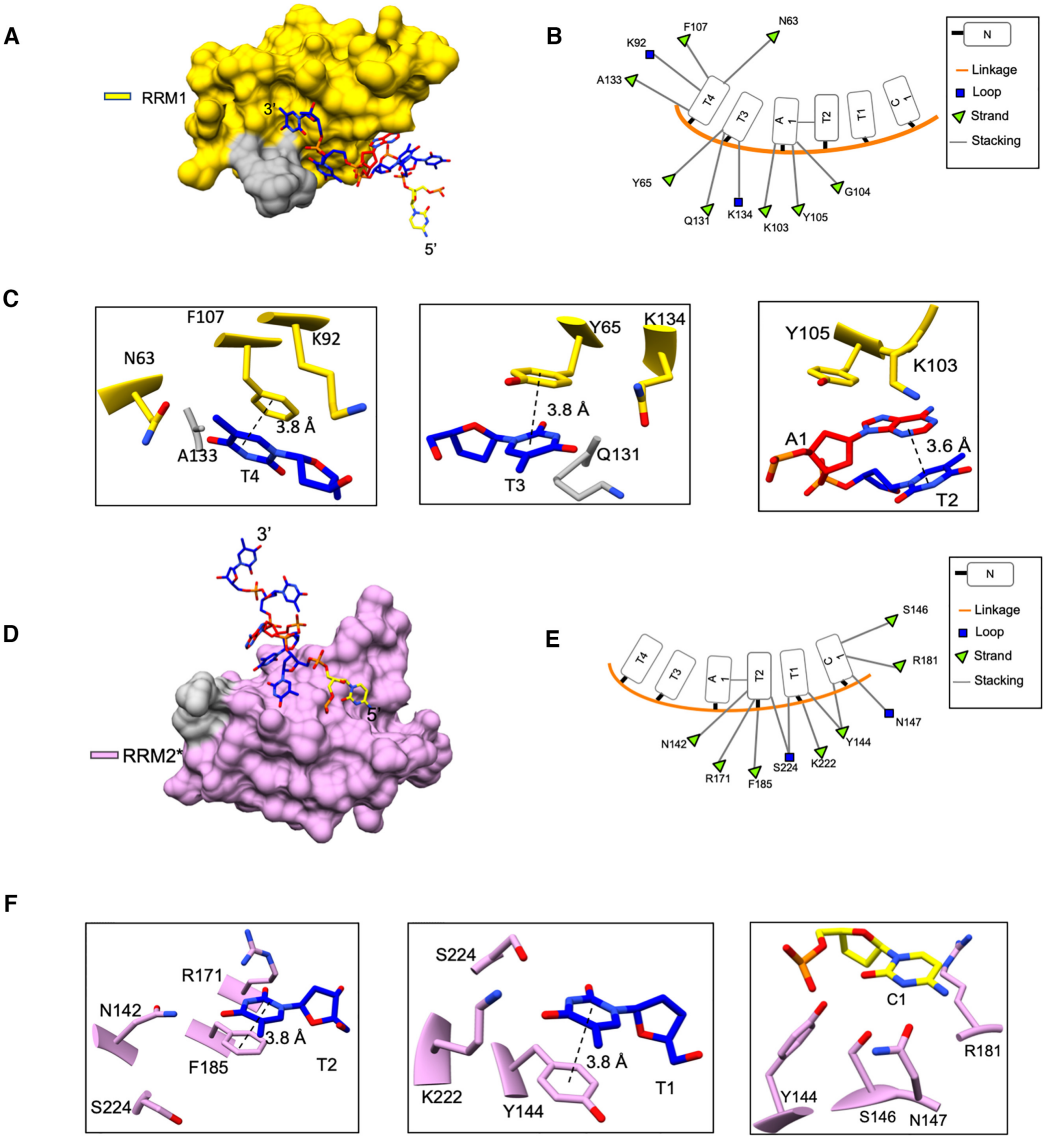
(**A**) Surface view of the RRM1 domain (yellow) and the 3′ terminal end of the DNA. (**B**) 2D representation of various types of molecular interactions between the amino acid residues of the RRM1 domain and the 3′ terminal end of the DNA with (**C**) Zoomed in views of the important interactions. (**D**) Surface view of the RRM2 domain of symmetry related molecule and the 5′ terminal end of the DNA with (**E**) 2D representation of various types of molecular interactions between the amino acid residues of the RRM2 domain and the 5′ terminal end of the DNA with (**F**) Zoomed in views of the important interactions.

To delineate the atomic interactions between the protein and DNA, we determined the crystal structure of RBMS1 protein (58–224) with the *c-myc* promoter consensus sequence TCTTATT (PDB id 6M75). The data collection and refinement statistics are presented in Table 1. The crystal asymmetric unit contained one molecule of protein and DNA each. RBMS1 adopted an open conformation wherein both domains were relatively far apart from each other. A comparison of the binding mode of the DNA to RBMS1 with a similar type of structure of HuD protein (23) (PDB id 1FXL) wherein the two RRM domains form a cleft in the shape of V to accommodate 11 nucleotide RNA sequence, suggested a deviation from the canonical binding. In the crystal structure, non-canonical binding mechanism of DNA was observed wherein the DNA binding spanned from one domain in one asymmetric unit to the other domain in the symmetry related molecule (Figure 3). Except for the 5′ terminal nucleotide, for which electron density could not be observed, 5 nucleotides of the DNA promoter consensus sequence TCTTATT made very specific contacts with the aromatic amino acid residues on the protein in a 5′ to 3′ direction from RRM2 to RRM1 domains. DNA promoter sequence's nucleotide bases T3, T4, T6 and T7 were involved in parallel π–π stacking with the amino acid residues Y144, F185, Y65 and F107, respectively (Figure 4), while A5 was involved in parallel displaced stacking with Y105. Y65 lies on the β1 strand of RRM1 and Y105 and F107 lie on the β3 strand of the RRM1 domain of RBMS1. The other two amino acids involved in stacking with DNA were positioned on the RRM2 domain of the protein with Y144 and F185 lying on the β1 and β3 strands of RRM2. These aromatic amino acid residues showed high CSP values in the solution-state NMR spectra (Supplementary Figure S5) and were located on RNP sites of these RRM domains. The interactions observed in the crystal structure were validated with the ITC data obtained from the mutations that were done in the aromatic amino acid residues on the RRM1 domain (Y105S, F107L), which proved to be utmost crucial for binding affinity as well as specificity of RBMS1. The mutations done on the RRM2 domain (F185V) showed they were important for governing the specificity of the binding only. The mutation of amino acid residue in the linker (Q138E) also affected the affinity and specificity of the binding of RBMS1 protein to the cognate DNA sequences (Supplementary Table S3). All these findings supported the molecular mechanism of DNA binding that was delineated from the crystal structure.

**Table 1.** X-Ray structural parameters for the RBMS1-TCTTATT complex structure. The Ramachandran plot statistics were obtained using PSVS

**PDB id**	6M75
**Data collection**	
Wavelength (Å)	1.54
Detector type	R-AXIS IV^++^
Oscillation (°)	0.5
Exposure (s)	240
No. of images	509
Software used for data processing	autoPROC
Space group	*P* 2_1_ 2_1_ 2
Cell dimensions	
*a*, *b*, *c* (Å)	83.68, 114.95, 27.43
α, β, γ (°)	90, 90, 90
<I/σ(I)>	11.9 (2.4)
Completeness (%)	99.7 (99.5)
CC_1/2_	0.995 (0.711)
Resolution (Å)	67.65–2.57 (2.61–2.57)
*R*_merge_	0.166 (0.998)
*R*_meas_ (%)	0.175 (1.052)
*R*_pim_	0.055 (0.328)
Redundancy	9.7 (10.0)
No. of unique reflections	9032 (428)
**Refinement**	
Resolution (Å)	19.66–2.57 (2.65–2.57)
*R*_free_ test set	900 reflections (10.00%)
*R*_work_/*R*_free_ (%)	0.211, 0.236
Total number of atoms	1466
Protein	1264
DNA	120
Solvent	32
Others	50
Overall CC (real space correlation)	0.83
Average *B*, all atoms (Å^2^)	48.9
Protein	48.1
DNA	78.7
R.m.s deviations	
Bond lengths (Å)	0.006
Bond angles (º)	1.09
Ramachandran plot	
Favoured/Allowed (%)	98.2/1.8
Rotamer outliers	0
C-beta outliers	0

The values in the parenthesis are the statistics for the last resolution shell.

### RRM domains undergo conformational and dynamic reorganization for DNA binding

The comparison of the free solution-state NMR structure of RBMS1 (58–224) with the complex structure of RBMS1 in bound form with the DNA showed the conformational change in RBMS1 upon binding to the DNA (Figure 3E). The opening of the 3_10_ helix in the linker region and movement of the RRM2 domain facilitated the binding of RBMS1 to the DNA. The overall RMSD of individual domain was calculated by separately overlaying individual domains in the X-ray structure of the complex and NMR structure of the free protein (Supplementary Figure S6a and b). As has been stated before, the mutagenesis studies of the linker residue Q138 proved the importance of repositioning of the linker to orient the domains in the correct pose.

NMR relaxation dynamics analysis showed that the binding of RBMS1 (58–224) protein with the promoter DNA sequence led to minor changes in the flexibility of the protein. This could be seen from the effective (isotropic) rotational correlation time, τ_c_, obtained from the model-free analysis of relaxation data, that increased to 11.0 ns from 8.8 ns upon formation of the complex of RBMS1 with c*-myc* promoter DNA. We calculated the average *T*1, *T*2, *S*^2^, *R*1/*R*2 and ^15^N–{^1^H} Het-nOe values for the RRM1 domain, RRM2 domain, and the linker in both free and bound forms (Table 2). There was an overall decrease in the *R*1/*R*2 ratio (Table 2, Supplementary Figure S3a) and an overall increase in the internal site-specific squared order parameter *S*^2^ (*S*^2^ = *Sf*^2^*Ss*^2^) of all the residues (Table 2, Supplementary Figure S3c). The values of τ_c_, *R*1 and *R*2 were within the range of values expected for a protein of size 18.6 kDa (24). The lower average of *R*1/*R*2 values of bound RBMS1 (0.12) compared to free RBMS1 (0.17) suggested that the rotational diffusion of the RBMS1 became slower in the bound form (Table 2, Supplementary Figure S3a). Subtle differences were seen as an increase in rigidity of the protein by the increase in *S*^2^ values of some residues at sites where the DNA interacts with the protein to form the complex; *S*^2^ for F185 residue increased from 0.89 to 0.98 and that of Y65 increased from 0.87 to 0.97 upon complex formation. However, as nOes show sensitivity to the local flexibility; an increase in the average nOe values from 0.64 to 0.72 for the linker suggested a decrease in its rapid local motion upon the complex formation (Table 2, Supplementary Figure S3b). The local motions of the individual domains also decreased upon complex formation, however, the change in nOe values was not as pronounced as was seen for the linker.

**Table 2.** Comparison of average *T*1, *T*2, *R*1/*R*2, *S*^2^ and ^15^N–{^1^H} Het-nOe values for the four constructs of RBMS1 protein in both free and DNA bound forms

Protein construct		*T*1 (s)	*T*2 (s)	*R*1/*R*2	*S* ^2^	^15^N–{^1^H} Het-nOe
RBMS1 (58–224)	Free	0.58	0.10	0.17	0.87	0.67
	Bound	0.63	0.08	0.12	0.93	0.71
RRM1 (58–132)	Free	0.57	0.10	0.17	0.87	0.67
	Bound	0.64	0.07	0.12	0.94	0.71
Linker (133–141)	Free	0.56	0.10	0.17	0.87	0.64
	Bound	0.62	0.08	0.12	0.93	0.72
RRM2 (142–224)	Free	0.60	0.09	0.16	0.87	0.67
	Bound	0.62	0.08	0.13	0.92	0.70

The calculated *R*1, *R*2, ^15^N–{^1^H} Het-nOe values, and the structural co-ordinates were used for calculating the overall rotational diffusion tensors using an anisotropic diffusion tensor model for the free RBMS1, free individual RRM1, and RRM2 domains and for the RBMS1, RRM1 and RRM2 domains in the complex form, using the program ROTDIF 1.1^17^ (Table 3). The residues whose local motions were pre-dominant, that is, the ones having ^15^N–{^1^H} nOe value less than 0.65 were excluded from the calculations. We observed uniform increase in the diffusion tensors and this possibly could have implication in the binding of DNA.

**Table 3.** The principal components of the anisotropic diffusion tensors of the different constructs of RBMS1 protein calculated using the program ROTDIF 1.1^17^

	RBMS1	RBMS1–TCTTATT	RRM1 free	RRM1 bound	RRM2 free	RRM2 bound
*D_x_* × 10^7^ s^–1^	0.96	1.40	0.94	1.41	0.91	1.33
*D_y_* × 10^7^ s^–1^	1.02	1.49	1.02	1.47	1.01	1.46
*D_z_* × 10^7^ s^–1^	1.07	1.77	1.08	1.70	1.12	1.92

*D_x_*, *D_y_* and *D_z_* denote the principal values of the anisotropic rotational diffusion tensors in the *x*, *y* and *z* directions, respectively.

In order to further understand the role of dynamics in DNA binding, we performed 1000 ns molecular dynamics (MD) simulations for free and TCTTATT DNA-bound states of RBMS1 protein. Both the free and DNA bound states of protein reached a state of equilibrium after 300 ns, as can be seen from the RMSD plots in Supplementary Figure S6c and d. In the case of the free protein, the RMSD deviation within the protein was within 14.0–16 Å towards the end of MD, while in the bound form it deviated in the range of 12.0–13.5 Å, showing, it essentially became more stable upon complex formation. The RMSF plot (Supplementary Figure S6e and f), which shows residue-wise deviation, depicted major changes in the last β4 strand and the preceding loop (amino acid residues 140–167) of RRM2 domain at the C-terminal end of the protein that showed a deviation of 6 Å, coming down from 12 Å in case of the free protein structure. Upon complex formation, the sheet of RRM2 domain came close to the 5′ terminal end of the DNA sequence, i.e. Y145 and F185 amino acid residues stacked parallel with the T3 and T4 nucleotide bases of the DNA (supplementary videos 1 and 2).

MD simulation videos clearly showed that the movement of RRM2 is crucial for the binding of RBMS1 to the DNA and also explain why the two domains could not be fixed with respect to each other in the solution-state NMR structure. The stoichiometry calculated in solution from ITC, NMR spectroscopy, and the MD simulation showed 1:1 binding wherein one protein molecule spanned across the promoter DNA consensus sequence with the RRM1 domain binding with the 3′ terminal end nucleotides of DNA, and the RRM2 domain binding with the 5′ terminal end nucleotides of the DNA sequence. Therefore, the changes in the orientation and positioning of the protein were required for protein to scan the bases and function at the specific promoter DNA sequence site only.

## DISCUSSION

DNA recognition by DNA binding proteins is a stochastic process, with little affinity differences towards the specific and non-specific DNA sequences (25). The thermodynamics and kinetics of DNA-protein interactions are the two major determinants that govern the specific and the non-specific binding (1–3). In this study, we report the DNA sequence recognition mechanism of a regulatory protein RBMS1 that stringently regulates proto-oncogene *c-myc* levels and presents the future for developing efficient cancer targeted gene therapy against *c-myc* proto-oncogene.

The crystal packing revealed unique structural features where the DNA binding spanned from one domain to the other domain of the symmetry related RBMS1 molecule. In the crystal structure, aromatic residues formed stacking interaction with the DNA bases. The role of aromatic residues has been implicated several times before in binding to the nucleic acid sequences inside the cell (26,27). The DNA did not bind to the protein in an extended form and a stacking network was seen between the T4 and A5 nucleotides of the DNA sequence (Figure 3B). The comparison between the free NMR and complex X-ray structures (Figure 3E) revealed that the RRM2 domain underwent a major change in its orientation in order to bind the DNA molecule. We observed that the two domains did not interact with each other and tumbled independently in the solution. It is known that the presence of multiple RRM domains in a protein increases its affinity to a stretch of nucleic acid as well as makes it possible for a nucleic acid binding protein to recognize a longer length of RNA/DNA nucleotides (28). For example, in the human HuD protein (23), two RRMs form a complex with the *c-fos* AU rich 11 nucleotide sequence, wherein the RRM1 domain and the interdomain linker binds with the U5 through U10, while the RRM2 accommodates two nucleotides U3 and U4; binding a total of 7 nucleotides in the 11-nucleotide sequence. Similarly, in CUGBP1 protein, RRM1 and 2 together binds to a 5 nucleotide long RNA sequence, where the RRM1 binds the U2 nucleotide and U3 through U6 nucleotides are bound by the RRM2 domain (29). Our calorimetric ITC studies showed that the DNA sequences of less than 6 nucleotides in length did not bind to RBMS1 protein, *in vitro*. Most RRM domain containing proteins, having two or more than two RRMs do not have all RRM domains participating in binding to the nucleic acid (23,28). This has been reported in the prp24 protein (30), wherein the first two RRMs out of total 4 RRMs in the protein are involved in binding with the U6 RNA. In our case, when we separated the two RRM domains of RBMS1, the RRM2 domain alone did not bind to any of the nucleotide sequences we titrated with it, and the binding of the RRM1 domain alone to the nucleic acid sequences decreased ∼10–18 fold. This is because T3 and T4 nucleotides were involved in the parallel π–π stacking with the Y144 and F185 amino acid residues, which are present on the RRM2 domain. Moreover, the proper positioning of domain 1 with respect to the DNA sequence was done with the help of the linker. Therefore, when the interaction of only the RRM1 domain was checked with the promoter DNA sequence, a 10–18 fold reduction in affinity of interaction was deemed justified. Hence, both the domains were necessary for RBMS1 to perform its designated function. We argue that the versatility shown by the RRM fold in binding to diverse sequences comes from the cooperation of more than one RRM domain to carry out its function. The binding of RBMS1 protein to the *c-myc* promoter DNA sequence decreased 10 times if the amino acid residue in the linker of the RBMS1 (58–224) was mutated (Supplementary Table S3). This re-instilled the importance of the linker in correctly positioning the two domains to bind with the promoter DNA sequence of *c-myc*.

We explored the process of DNA scanning by RBMS1 protein by mutating the DNA sequence by changing one or more bases. We wondered if the stacking interaction that was seen between the bases of the promoter nucleotide sequence was also a factor in deciding the orientation/pose of RBMS1 and its specificity, or other factors were at play too. Our results revealed that within all the sequences that were used for the thermodynamics calculations, very little difference was seen in the affinity between the specific and non-specific sequences. However, entropy is speculated to play a major role in the modulation of the specificity of interactions. It was observed that the change in entropy of RBMS1’s interaction with TCTTATT was the lowest amongst all the sequences that were analysed thermodynamically using ITC.

The overall dynamics in the supplementary videos 1 and 2 showed that the orientation of the RRM2 domain changed, and it became ordered when it came close to the 5′ terminal of DNA. There were minor changes in the flexibility of the RBMS1 protein upon binding but further experimental investigations will help in establishing the role of dynamics in recognition of the *c-myc* promoter by the RBMS1 protein. Site directed mutagenesis studies showed that the mutation of aromatic residues on the RRM1 domain had a larger impact on the binding of RBMS1 to the *c-myc* promoter, which complemented the structural finding that the RRM2 domain was mainly involved in correctly positioning the RRM1 domain onto the DNA. The complementarity in the conformation of the binding sequence's nucleotides and the corresponding conformation of the RRM domain could be a crucial factor in governing the sequence specificity and the designated function.

To summarize, in this study we have determined the structural and thermodynamics basis of *c-myc* promoter DNA recognition by RBMS1 protein. Finally, more such structural and thermodynamics studies aimed at similar DNA-protein complexes need to be done to get more mechanistic insights, and direct better designing of the future anti-gene therapies.

## DATA AVAILABILITY

Solution structures of free RBMS1 and crystal structure of TCTTATT DNA-bound form have been deposited in the Protein Data Bank (PDB) under accession numbers PDB id: 7C36 and PDB id: 6M75, respectively. The corresponding deposition of NMR resonance assignments in the BioMagResBank (BMRB) have been made under accession numbers BMRB id: 36354. All other data are available from the corresponding author on a reasonable request.

## Supplementary Material

gkab363_Supplemental_Files

## References

[1] Murphy F.C.M. Nonsequence-specific DNA recognition: a structural perspective. Structure. 2000; 8:R83–R89. 10801483 10.1016/s0969-2126(00)00126-x

[2] Iwaharaa J. , Zandarashvili L., Kemmea C.A., Esadze A. NMR-based investigations into target DNA search processes of proteins. Methods. 2018; 148:57–66. 29753002 10.1016/j.ymeth.2018.05.004 PMC6133758

[3] Iwahara J. , Zweckstetter M., Clore G.M. NMR structural and kinetic characterization of a homeodomain diffusing and hopping on nonspecific DNA. Proc. Natl. Acad. Sci. U.S.A. 2006; 103:15062–15067. 17008406 10.1073/pnas.0605868103 PMC1622777

[4] Alberts B. , Johnson A., Lewis J., Raff M., Roberts K., Walter P. Molecular Biology of the Cell 173–236. Garland Science.

[5] Wieczor M. , Czub J. How proteins bind to DNA: target discrimination and dynamic sequence search by the telomeric protein TRF1. Nucleic Acids Res. 2017; 45:7643–7654. 28633355 10.1093/nar/gkx534 PMC5737604

[6] Marcu K.B. , Patel A.J., Yang Y. Differential regulation of the c-MYC P1 and P2 promoters in the absence of functional tumor suppressors: implications for mechanisms of deregulated MYC transcription. Curr. Top. Microbiol. Immunol. 1997; 224:47–56. 9308227 10.1007/978-3-642-60801-8_4

[7] Nagai K. , Oubridge C., Ito N., Avis J., Evans P. The RNP domain: a sequence-specific RNA-binding domain involved in processing and transport of RNA. Trends Biochem. Sci. 1995; 20:235–240. 7543225 10.1016/s0968-0004(00)89024-6

[8] Niki T. , Galli I., Ariga H., Iguchi-Ariga S.M. MSSP, a protein binding to an origin of replication in the *c-myc* gene, interacts with a catalytic subunit of DNA polymerase alpha and stimulates its polymerase activity. FEBS Lett. 2000; 475:209–212. 10869558 10.1016/s0014-5793(00)01679-3

[9] Negishi Y. , Nishita Y., Saëgusa Y., Kakizaki I., Galli I., Kihara F., Tamai K., Miyajima N., Iguchi-Ariga S.M., Ariga H. Identification and cDNA cloning of single-stranded DNA binding proteins that interact with the region upstream of the human *c-myc* gene. Oncogene. 1994; 9:1133–1143. 8134115

[10] Takai T. , Nishita Y., Iguchi-Ariga S.M., Ariga H. Molecular cloning of MSSP-2, a *c-myc* gene single-strand binding protein: characterization of binding specificity and DNA replication activity. Nucleic. Acids. Res. 1994; 22:5576–5581. 7838710 10.1093/nar/22.25.5576 PMC310119

[11] Niki T. , Izumi S., Saëgusa Y., Taira T., Takai T., Iguchi-Ariga S.M., Ariga H. MSSP promotes ras/myc cooperative cell transforming activity by binding to c-Myc. Genes Cells. 2000; 5:127–141. 10672043 10.1046/j.1365-2443.2000.00311.x

[12] Keller R. The Computer Aided Resonance Assignment Tutorial. 2004; CANTINA Verlag, Goldau.

[13] Bax A. , Grzesiek S. Methodological advances in protein NMR. Acc. Chem. Res. 1993; 26:131–138.

[14] Shen Y. , Bax A. Protein backbone and sidechain torsion angles predicted from NMR chemical shifts using artificial neural networks. J. Biomol. NMR. 2013; 56:227–241. 23728592 10.1007/s10858-013-9741-y PMC3701756

[15] Guntert P. Automated NMR structure calculation with CYANA. Methods Mol. Biol. 2004; 278:353–378. 15318003 10.1385/1-59259-809-9:353

[16] Maier J.A. , Martinez C., Kasavajhala K., Wickstrom L., Hauser K.E., Simmerling C. ff14SB: improving the accuracy of protein side chain and backbone parameters from ff99SB. J. Chem. Theory Comput. 2015; 11:3696–3713. 26574453 10.1021/acs.jctc.5b00255 PMC4821407

[17] Berlin K. , Longhini A., Dayie T.K., Fushman D. Deriving Quantitative Dynamics Information for Proteins and RNAs using ROTDIF with a Graphical User Interface. J. Biomol. NMR. 2013; 57:333–352. 24170368 10.1007/s10858-013-9791-1 PMC3939081

[18] Vonrhein C. , Flensburg C., Keller P., Sharff A., Smart O., Paciorek W., Womack T., Bricogne G. Data processing and analysis with the autoPROC toolbox. Acta Crystallogr. D. Biol. Crystallogr. 2011; 67:293–302. 21460447 10.1107/S0907444911007773 PMC3069744

[19] Adams P.D. , Afonine P.V., Bunkóczi G., Chen V.B., Davis I.W., Echols N., Headd J.J., Hung L.W., Kapral G.J., Grosse-Kunstleve R.W. et al. PHENIX: a comprehensive Python-based system for macromolecular structure solution. Acta Crystallogr. D. Biol. Crystallogr. 2010; 66:213–221. 20124702 10.1107/S0907444909052925 PMC2815670

[20] Emsley P. , Cowtan K. Coot: model-building tools for molecular graphics. Acta Crystallogr. D. Biol. Crystallogr. 2004; 60:2126–2132. 15572765 10.1107/S0907444904019158

[21] Pettersen E.F. , Goddard T.D., Huang C.C., Couch G.S., Greenblatt D.M., Meng E.C., Ferrin T.E. UCSF Chimera – a visualization system for exploratory research and analysis. J. Comput. Chem. 2004; 25:1605–1612. 15264254 10.1002/jcc.20084

[22] Roos K. , Wu C., Damm W., Reboul M., Stevenson J.M., Lu C., Dahlgren M.K., Mondal S., Chen W., Wang L. et al. OPLS3e: extending force field coverage for drug-like small molecules. J. Chem. Theory Comput. 2019; 15:1863–1874. 30768902 10.1021/acs.jctc.8b01026

[23] Wang X. , Tanaka Hall T.M. Structural basis for recognition of AU-rich element RNA by the HuD protein. Nat. Struct. Biol. 2001; 8:141–145. 11175903 10.1038/84131

[24] Rossi P. , Swapna G.V.T., Huang Y.J., Aramini J.M., Anklin C., Conover K., Hamilton K., Xiao R., Acton T.B., Ertekin A. et al. A microscale protein NMR sample screening pipeline. J. Biomol. NMR. 2010; 46:11–22. 19915800 10.1007/s10858-009-9386-z PMC2797623

[25] Siggers T. , Gordan R. Protein-DNA binding: complexities and multi-protein codes. Nucleic. Acids. Res. 2014; 42:2099–2111. 24243859 10.1093/nar/gkt1112 PMC3936734

[26] Clery A. , Blatter M., Allain F.H. RNA recognition motifs: boring? Not quite. Curr. Opin. Struct. Biol. 2008; 18:290–298. 18515081 10.1016/j.sbi.2008.04.002

[27] Daubner G.M. , Clery A., Allain F.H. RRM-RNA recognition: NMR or crystallography…and new findings. Curr. Opin. Struct. Biol. 2013; 23:100–108. 23253355 10.1016/j.sbi.2012.11.006

[28] Afroz T. , Cienikova Z., Clery A., Allain F.H.T. One, two, three, four! How multiple RRMs read the genome sequence. Methods Enzymol. 2015; 558:235–278. 26068744 10.1016/bs.mie.2015.01.015

[29] Teplova M. , Song J., Gaw H.Y., Teplov A., Patel D.J. Structural insights into RNA recognition by the alternate-splicing regulator CUG-binding protein 1. Structure. 2010; 18:1364–1377. 20947024 10.1016/j.str.2010.06.018 PMC3381513

[30] Bae E. , Reiter N.J., Bingman C.A., Kwan S.S., Lee D., Phillips G.N., Butcher S.E., Brow D.A. Structure and interactions of the first three RNA recognition motifs of. splicing factor Prp24. J. Mol. Biol. 2007; 367:1447–1458. 17320109 10.1016/j.jmb.2007.01.078 PMC1939982

