# Supplementary material for: Hinge like domain motion facilitates human RBMS1 protein binding to proto-oncogene c-myc promoter
Source: Nucleic Acids Res. 2021 May 17;49(10):5943–55. doi: 10.1093/nar/gkab363 (PMC8191779; doi:10.1093/nar/gkab363)
Supplement: gkab363_Supplemental_Files [file gkab363_supplemental_files.zip › NAR SUPPLEMENTARY.pdf]

Hinge like domain motion facilitates human RBMS1 protein binding to proto-oncogene *c-myc* promoter

Priyanka Aggarwal and Neel Sarovar Bhavesh**
Transcription Regulation group, International Centre for Genetic Engineering and Biotechnology (ICGEB), Aruna Asaf Ali Marg, New Delhi, India 110067

*To whom correspondence should be addressed. E-mail: priyanka.pdm@gmail.com, neelsb@icgeb.res.in

SUPPLEMENTARY DATA FIGURES AND TABLES

SUPPLEMENTARY TABLE S1: NMR structural parameters for the energy minimized 20 conformers of RBMS1 (58-224) calculated using CYANA 3.98.13. RMSDs were evaluated using UCSF-Chimera and Ramachandran plot statistics were obtained using PSVS.

Average target function ($Å^2$)	1.72 ± 0.17
NMR distance and dihedral constraints	2185
**RMSD values (Å)**	
N-terminal RRM1 domain (Residues 58-132)	0.797
C-terminal RRM2 domain (Residues 142-224)	0.917
**Ramachandran plot statistics**	
Residues in most favorable regions (%)	94.3
Residues in allowed regions (%)	5.3
Residues in generously allowed regions (%)	0
Residues in disallowed regions (%)	0.3
RMS deviation for bond angles (°)	1.9
RMS deviation for bond lengths (Å)	0.012

SUPPLEMENTARY TABLE S2: ITC data to determine sequence specificity for RBMS1 (58-224) protein.

DNA Sequence	K_d (µM)	ΔS (cal/mol/deg)	ΔH (kcal/mol)
TCTCTTATGCGGTTGAATAGT	2.6	-79.7	-31.90
TCTTATT	3.84	-70.5	-28.89
TGTTATT	15.2	-44.7	-20.23
TCGTATT	7.7	-38.7	-18.80
TGGTATT	6.7	-29.4	-16.09
TCATATT	11.9	-40.8	-19.20
TCTTATG	19.3	-25.4	-14.20
TATAATT	37.0	-41.5	-18.70
TGGTTTT	4.04	-32.0	-17.17
TGGAATT	20.96	-13.3	-10.51
TGGTATC	6.2	-31.5	-16.77
TGGTGTT	16.12	-19.8	-12.65
TGGTACT	15.94	-14.9	-11.18
TGGTAAT	9.6	-12.1	-17.00
TGGGATT	36.36	-11.7	-18.60
TCGGATG	35.46	-6.2	-0.33
TCCCATT	8.2	-15.2	-27.00
TGGTCAT	17.51	-14.5	-26.10
TGCTATT	8.69	-47.0	-21.27
TGTTATT	13.29	-54.5	-23.30
GGTAAT	14.88	-45.8	-20.59
TTTTT	79.36	-18.9	-11.42
GTTAT	81.3	-33.1	-15.70
CTTAT	65.3	-18.8	-11.40
CTTAA	65.3	-18.8	-11.40
CTTTA	55.5	-16.4	-34.80
TTAT	No binding		
GTAT	No binding		
ATAT	No binding		

SUPPLEMENTARY TABLE S3: ITC data to determine the effect of mutations in amino acid residues of RBMS1 (58-224) protein on affinity and specificity for the three selected oligonucleotide sequences.

Protein mutant	DNA Sequence	K_d (µM)	ΔS (cal/mol/deg)	ΔH (kcal/mol)
Y105S	TCTTATT	No binding		
	TGGTATT	151.05	-9.9	-8.31
	TGGTTTT	418.00	-90.6	-3.21
F107L	TCTTATT	141.60	-27.2	-13.57
	TGGTATT	182.40	-30.2	-14.36
	TGGTTTT	83.33	-34.6	-11.06
Q138E	TCTTATT	13.94	-58.5	-24.46
	TGGTATT	17.57	-32.1	-16.33
	TGGTTTT	18.05	-25.9	-14.44
F185V	TCTTATT	6.02	-61.2	-25.80
	TGGTATT	23.15	-56.1	-23.43
	TGGTTTT	21.97	-47.0	-20.72

SUPPLEMENTARY TABLE S4: Primer sequences used for protein mutagenesis.

**Y105S**	**Forward primer**	**5′** CACCAATAAATGCAAAGGCTCCGGCTTTGTGGATTTTGATAG **3′**
	**Reverse primer**	**5′** CTATCAAAATCCACAAAGCCGGAGCCTTTGCATTTATTGGTG **3′**
**F107L**	**Forward primer**	**5′** GCAAAGGCTATGGCTTAGTGGATTTTGATAGTC **3′**
	**Reverse primer**	**5′** GACTATCAAAATCCACTAAGCCATAGCCTTTGC **3′**
**Q134E**	**Forward primer**	**5′** CAAAACAGCAAGAAGAGGATCCGACAAACC **3′**
	**Reverse primer**	**5′** GGTTTGTCGGATCCTCTTCTTGCTGTTTTG **3′**
**F185V**	**Forward primer**	**5′** CGTGGTGTGGGTGTTGCACGTATGG **3′**
	**Reverse primer**	**5′** CCATACGTGCAACACCCACACCACG **3′**

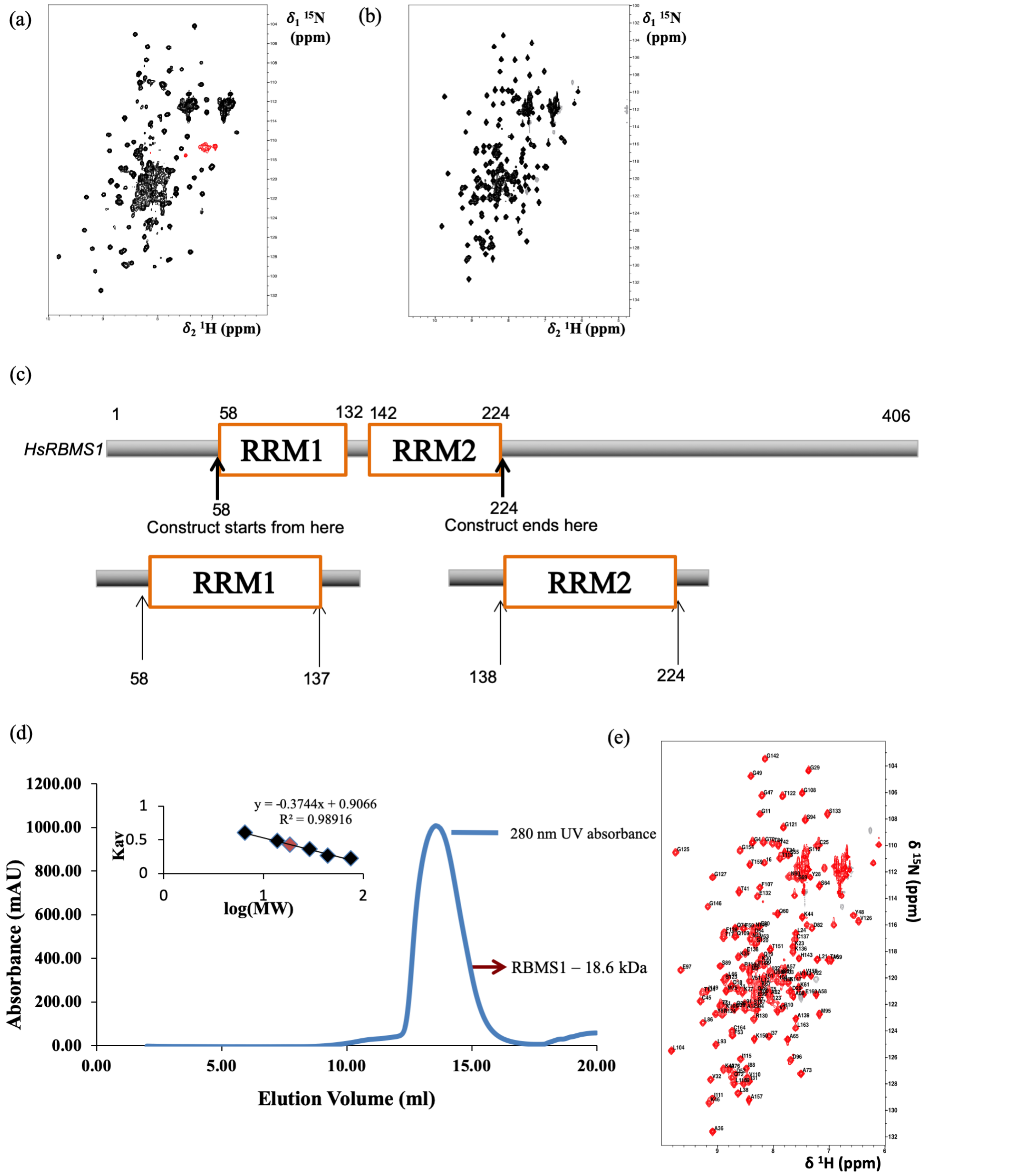

SUPPLEMENTARY FIGURE S1: (a) 2D [^{15}N,^{1}H] HSQC spectrum of RBMS1 with construct boundaries from 58 to 219 amino acid residues. **(b)** 2D [^{15}N,^{1}H] HSQC spectrum of RBMS1 with construct boundaries from 58 to 224 amino acid residues. **(c)** Schematic of construct boundaries of 3 constructs of RBMS1 used in the current study. Domain boundaries are shown as red boxes while the grey bar represents the unstructured region of the protein. **(d)** Size exclusion chromatogram of RBMS1 protein (58-224). Inset – the calibration profile for the GE superdex 75 size exclusion column is shown. **(e)** The 2D [^{15}N,^{1}H] HSQC NMR spectrum of RBMS1 (58-224) with sequence-specific resonance assignments of backbone amides marked.

(a)

```
                                                      RRM1
RBMS1    --------------PSTTSSN----NNSSSSSNSGWDQL---SKTNLYIRGLPP-HTTDQ    76
RBM14    --------------------------------------------MKIFVGNVDGADTTPE    16
DND1     ------------------VNG----QRKYGGPPPGWVGSPPPAGSEVFIGRLPQ-DVYEH    72
HNRNPD   SGGTEGGSAESEGAKIDASKNEEDEGHSNSSPRHSEAATAQREEWKMFIGGLSW-DTTKK    111

                                     RRM1
RBMS1    DLVKLCQPYGKIVSTKAILDKTTNKCKGYGFVDFDSPAAAQKAVSA----LKASGVQA--    130
RBM14    ELAALFAPYGTVMSCA--------VMKQFAFVHMRENAGALRAIEA----LHGHELRPGR    64
DND1     QLIPLFQRVGRLYEFRLMM-TFSGLNRGFAYARYSSRRGAQAAIAT----LHNHPLRPSC    127
HNRNPD   DLKDYFSKFGEVVDCTLKLDPITGRSRGFGFVLFKESESVDKVMDQKEHKLNGKVIDPKR    171

                                         RRM2
RBMS1    ----QMAKQQEQDPTNLYISNLPLSMDEQELENMLKPFGQVISTRILRDS-SGTSRGVGF    185
RBM14    ALVVEMSRPRPLNTWKIFVGNVSAACTSQELRSLFERRGRVI-ECDVV-------KDYAF    116
DND1     PLLVCRSTEKCE----LSVDGLPPNLTRSALLLALQPLGPGLQEARLLPSPGPAPGQIAL    183
HNRNPD   AKAMK----TKEPVKKIFVGGLSPDTPEEKIREYFGGFGEVESIELPMDNKTNKRRGFCF    227

                                RRM2
RBMS1    ARMESTEK---CEAVI----GHFNGK---FIKTPPGVSAPTE----PLLCKFADGGQKKR    231
RBM14    VHMEK---EADAKAAI----AQLNGK---EVKGKRIN------------VELST--KGQK    152
DND1     LKFSSHRAAAMAKKALVEGQSHLCGE---QVAVEWLK------------PDLKQRLRQQL    228
HNRNPD   ITFKEEEP---VKKIM-EKKYHNVGLSKCEIKVAMSKEQYQQQQQWGSRGGFAGRARGRG    283
```

(b)

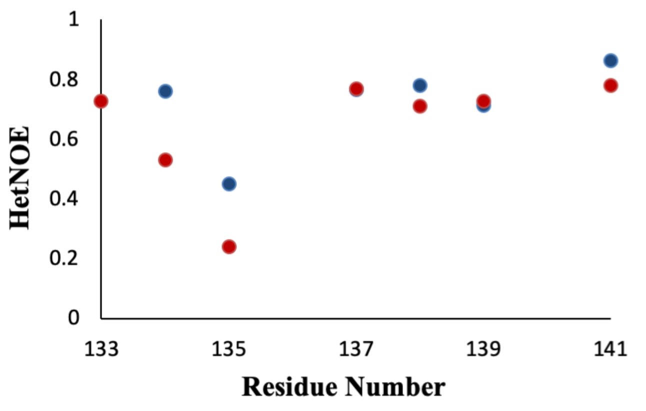

(c)

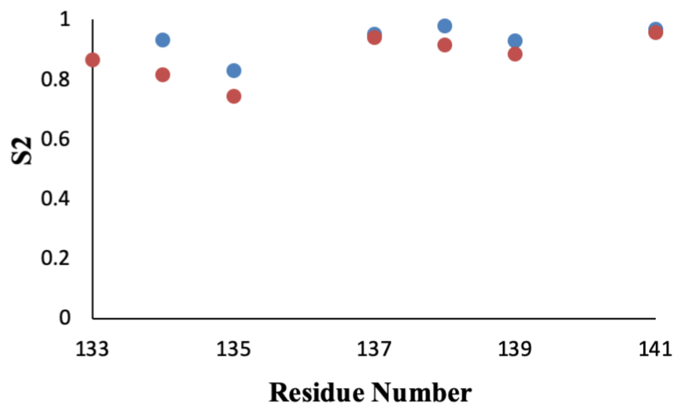

SUPPLEMENTARY FIGURE S2: (a) ClustalW sequence alignment of RBMS1 protein with the other proteins of the RRM family. The RRM domains are marked and the RNP sites have been marked in red (RNP2 site) and blue (RNP1 site) on each of the RRM domains. **(b)** ^{15}N-{^{1}H} Het-nOe for backbone amides of linker residues of RBMS1 in free form (red) and bound to TCTTATT DNA (blue). **(c)** The site-specific overall squared order free parameter to compare the difference in internal dynamics of linker residues of RBMS1 (red) upon forming complex with the DNA sequence (blue) is shown.

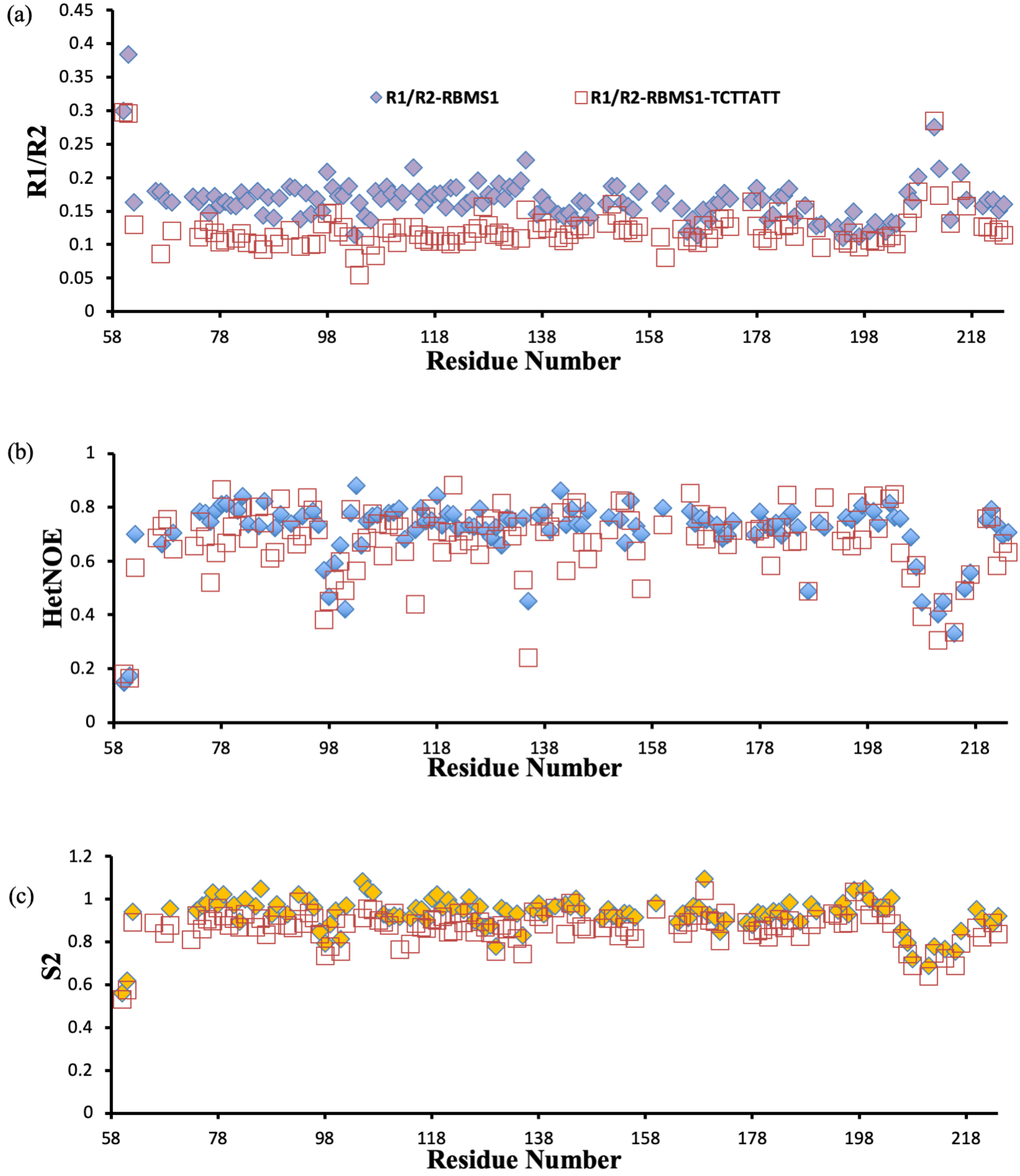

SUPPLEMENTARY FIGURE S3: (a) The comparison of internal dynamics of free and complex structure of RBMS1 *via* site-specific R1/R2 plots. **(b)** ^{15}N-{^{1}H} Het-nOe for backbone amides of RBMS1 in free form (red) and bound to TCTTATT DNA (blue). **(c)** The derived site-specific overall squared order free parameter (S^2) to compare the difference in internal dynamics of RBMS1 (red) upon forming complex with the DNA sequence (blue boxes filled in yellow) is shown.

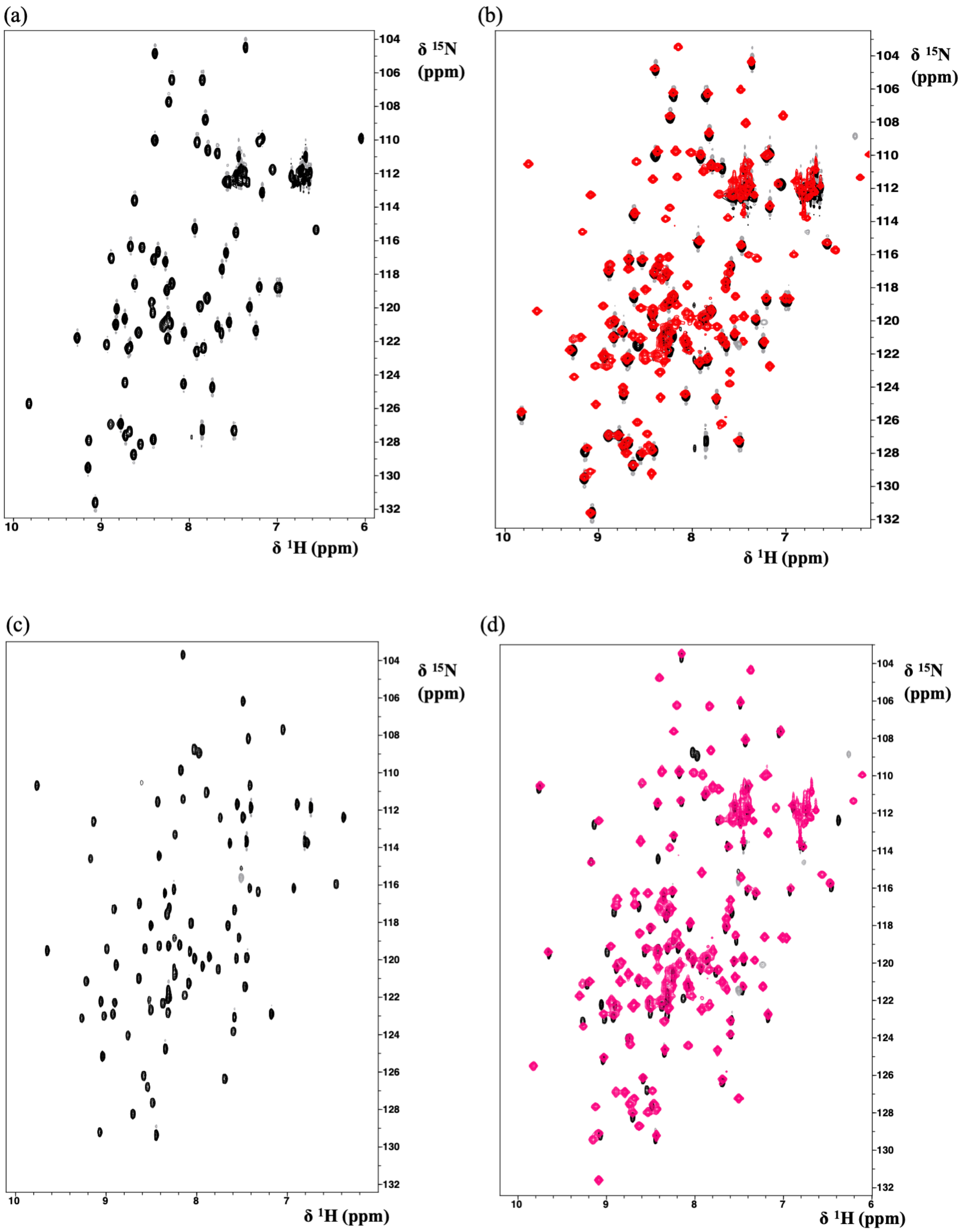

SUPPLEMENTARY FIGURE S4: (a) The 2D [^{15}N,^{1}H] HSQC spectrum of RRM1 domain (residues 58-137) **(b)** Overlay of 2D [^{15}N,^{1}H] HSQC spectra of RRM1 domain (residues 58-137) (black) and RBMS1 (58-224) (red). **(c)** The 2D [^{15}N,^{1}H] HSQC spectrum of RRM2 domain (residues 138-224) **(d)** Overlay of 2D [^{15}N,^{1}H] HSQC spectra of RRM2 domain (residues 138-224) (black) and RBMS1 (58-224) (pink).

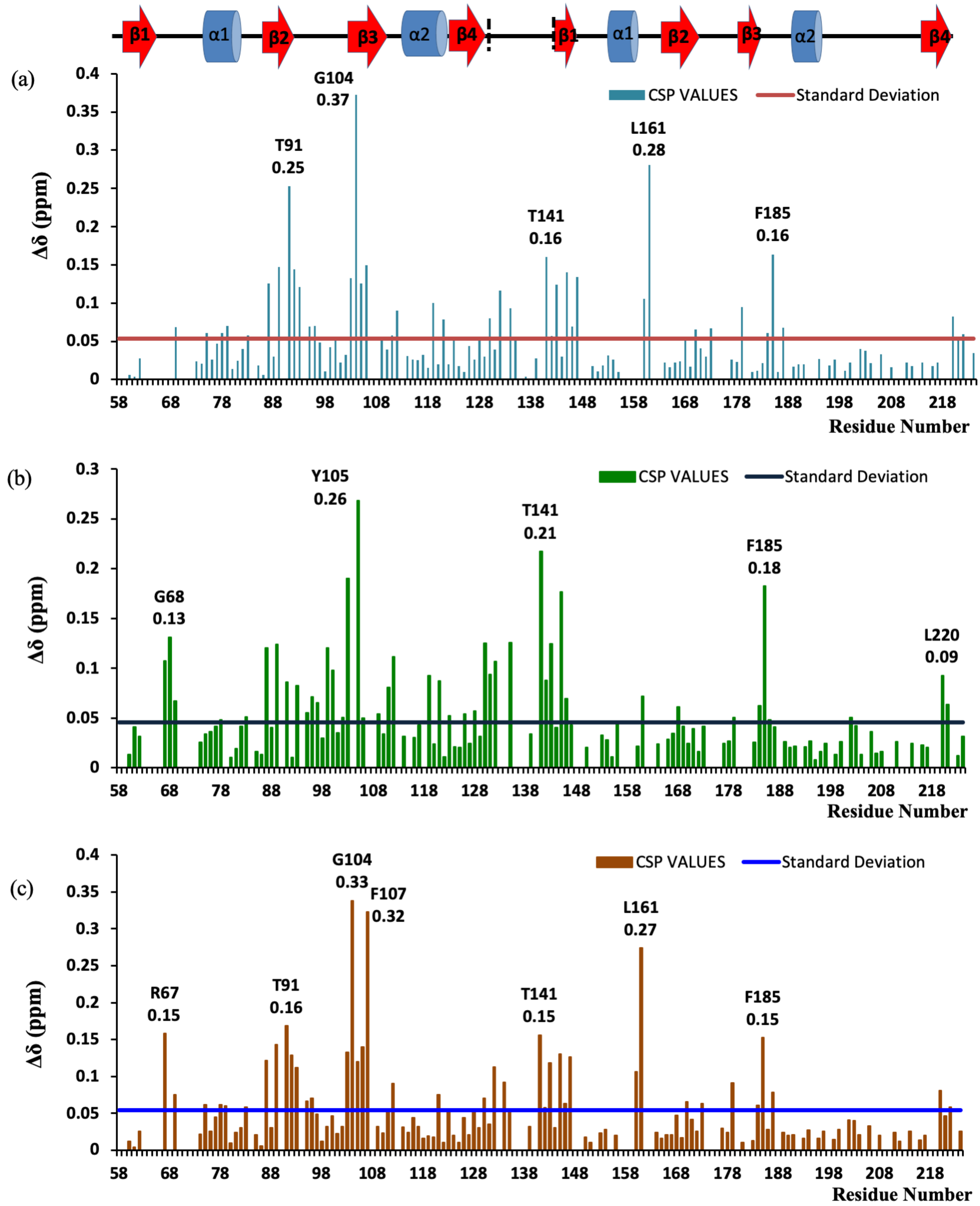

SUPPLEMENTARY FIGURE S5: Chemical Shift Perturbation (CSP) plots of selected DNA sequences with RBMS1 protein (58-224). The secondary structure elements of the protein are shown on the top, and start and end residues of the linker between the two domains are marked with dotted lines. Significantly perturbed residues are labeled. **(a)** CSP plot with the DNA sequence TGGTATT **(b)** CSP plot with the DNA sequence TGGTTTT **(c)** CSP plot with the DNA sequence TCTTATT.

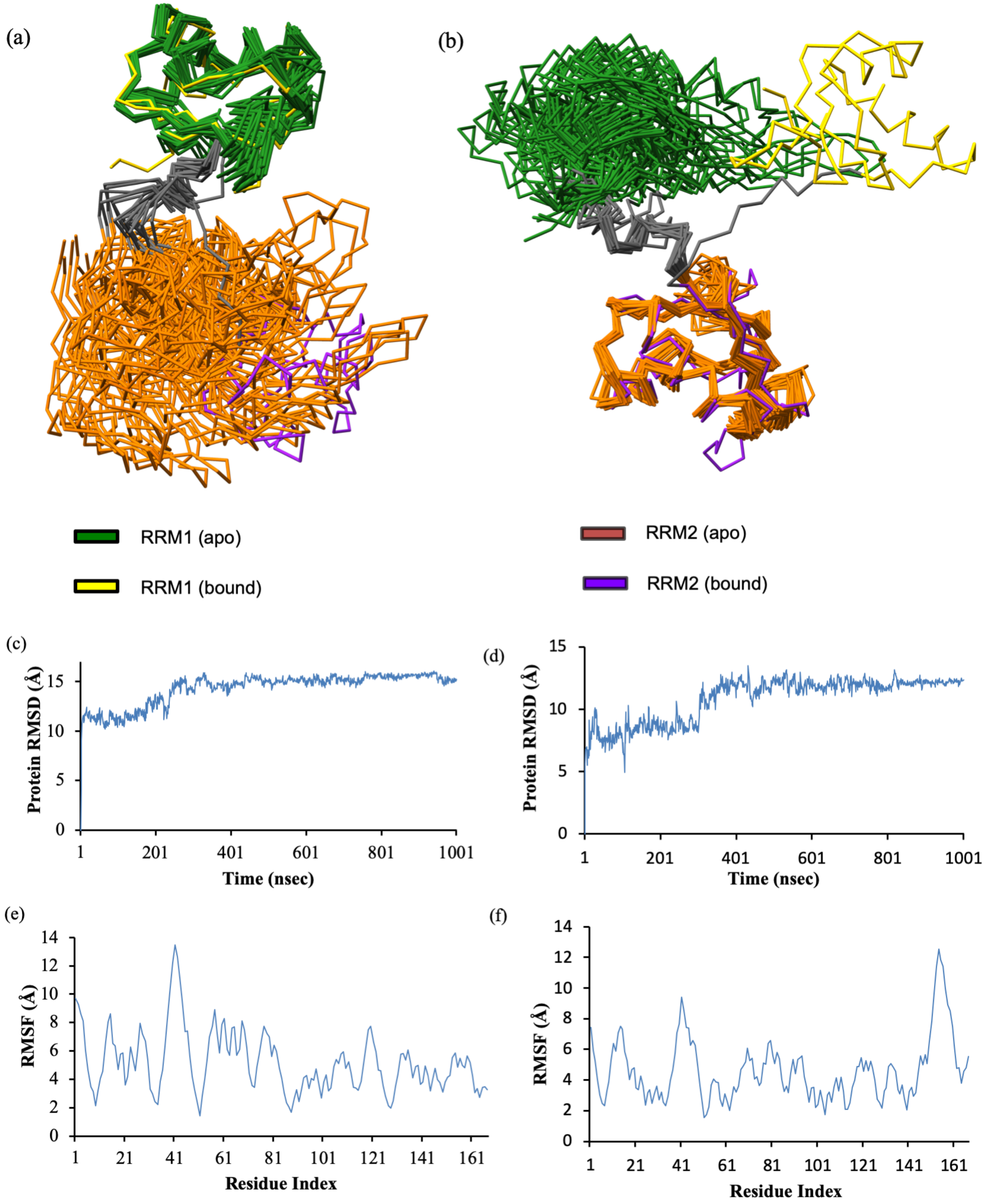

SUPPLEMENTARY FIGURE S6: (a) Superimposition of the backbone C-alpha atoms of solution NMR structure and complex crystal structure aligned with respect to the RRM1 domain (residues 58-132) with a RMSD of 1.07 Å. **(b)** Superimposition of the backbone C-alpha atoms of solution NMR structure and complex crystal structure aligned with respect to the RRM2 domain (residues 142-224) with a RMSD of 1.13 Å. Overall 1 µs dynamics is shown by **(c)** RMSD plot of RBMS1-TCTTATT complex **(d)** RMSD plot of RBMS1. **(e)** RMSF plot of RBMS1-TCTTATT complex. **(f)** RMSF plot of RBMS1
